# Reducing conditions are the key for efficient production of active ribonuclease inhibitor in *Escherichia coli*

**DOI:** 10.1186/1475-2859-10-31

**Published:** 2011-05-10

**Authors:** Juozas Šiurkus, Peter Neubauer

**Affiliations:** 1 Thermo Fisher Scientific (formerly Fermentas) V. A. Graiciuno 8, LT-02241 Vilnius Lithuania; 2 Laboratory of Bioprocess Engineering, Department of Biotechnology, Technische Universität Berlin, Ackerstr. 71-76, ACK24, D-13355 Berlin, Germany

## Abstract

**Background:**

The eukaryotic RNase ribonuclease/angiogenin inhibitors (RI) are a protein group distinguished by a unique structure - they are composed of hydrophobic leucine-rich repeat motifs (LRR) and contain a high amount of reduced cysteine residues. The members of this group are difficult to produce in *E. coli *and other recombinant hosts due to their high aggregation tendency.

**Results:**

In this work dithiothreitol (DTT) was successfully applied for improving the yield of correctly folded ribonuclease/angiogenin inhibitor in *E. coli *K12 periplasmic and cytoplasmic compartments. The feasibility of the *in vivo *folding concepts for cytoplasmic and periplasmic production were demonstrated at batch and fed-batch cultivation modes in shake flasks and at the bioreactor scale.

Firstly, the best secretion conditions of RI in the periplasmic space were evaluated by using a high throughput multifactorial screening approach of a vector library, directly with the Enbase fed-batch production mode in 96-well plates. Secondly, the effect of the redox environment was evaluated in isogenic *dsbA^+ ^*and *dsbA*^- ^strains at the various cultivation conditions with reducing agents in the cultivation medium. Despite the fusion to the signal peptide, highest activities were found in the cytoplasmic fraction. Thus by removing the signal peptide the positive effect of the reducing agent DTT was clearly proven also for the cytoplasmic compartment. Finally, optimal periplasmic and cytoplasmic RI fed-batch production processes involving externally added DTT were developed in shake flasks and scaled up to the bioreactor scale.

**Conclusions:**

DTT highly improved both, periplasmic and cytoplasmic accumulation and activity of RI at low synthesis rate, i.e. in constructs harbouring weak recombinant synthesis rate stipulating genetic elements together with cultivation at low temperature. In a stirred bioreactor environment RI folding was strongly improved by repeated pulse addition of DTT at low aeration conditions.

## Background

*Escherichia coli *is the most widely used host for recombinant protein production. Aggregation of the target protein in *E. coli *is a common phenomenon which is a consequence of the inability of the host's folding machinery to cope with the rapidly accumulating target protein folding and/or to facilitate efficient stabilization of SH groups, or to contribute to the formation and/or reorganization of correct disulfide bonds.

Contrary to most cases reported in literature, which focussed on the enhancement of disulfide bond formation in recombinant proteins by modulating the redox situation, we found it challenging to improve the folding of eukaryotic ribonuclease inhibitor RI (~49 kDa) which is characterised by a high amount of reduced cysteins, which are vital for the function of the protein. Our model protein - RI, shows a homology of 79-82% to the well characterized RNase ribonuclease/angiogenin inhibitors from human (hRI), rat (rRI), mouse (mRI) and porcine (pRI). The members of the ribonuclease inhibitor group represent a specific subfamily within the large group of proteins with a very special protein fold - the leucine-rich repeat (LRR) proteins [1]. LRR proteins share very interesting features which makes them a unique group of proteins. RI has an unusual non-globular flexible horseshoe like structure, which is very conserved between different species. The core of RI molecules is composed of hydrophobic 15-16 LRR motifs. Each of the LRR's consists of a structural unit of 28 to 29 amino acids forming an α-helix and β-strand connected by loops [2]. RI has a very high leucine content (18%), but also contains 30-32 cysteine residues (6.5-7%). In difference to other LRR motif containing proteins where the cysteins are structural units (see e.g. [3]), all cysteins in RI are reduced which is very important for activity, i.e. substate interaction. Oxidation of free SH groups in RI is highly cooperative and leads to inactivation and even denaturation [2,4].

Production of RI has been a challenge due to its flexible structure, repetetive amino acids and reduced cysteins. Thus so far reported RI production attempts in the yeast *Saccharomyces cerevisiae *[5] and in *E. coli *[6,7] resulted in a low overall yield either due to a low production level and/or high RI insolubility, respectively. So far about 10 mg of active porcine RI (pRI) per liter of culture medium was produced by using the P_trp _promoter in the *E. coli *host [7].

Recently, after high throughput multifactorial screening of an *E. coli *plasmid vector library which contained different promoters, ribosome binding sites and various fusion partners, we indentified factors which allowed us to obtain high amounts of soluble RI only in fusion with a MBP tag [8]. A fed-batch process was developed with the most favourable vector yielding about 800 mg of MBP-RI fusion protein per litre of mineral salt medium, which corresponds to 425 mg of RI. A similar result were recently published by Guo et al. [9]. The authors, in agreement with our earlier results, found the MBP tag the most suitable partner for soluble RI accumulation [9]. A drawback of this *fusion however is that the inhibitory activity of the MBP*-RI fusion *towards RNaseA is *12-fold decreased compared to untagged RI [8]. However, despite high level production, all other cytoplasmic constructs containing untagged RI or RI fused to GST, SUMO and thioredoxin (TRX) showed high aggregation levels, independently from the transcriptional or translational control units which were varied in the constructs [8]. Based on these results we suspected that the above mentioned molecular features of RI - their sensitivity to the redox environment and hydrophobicity, or a combination of both, could be the main causes stipulating aggregation in the *E. coli *cells.

In this work we applied RI production strategies which were more focused on gaining knowledge and understanding about the significance of reduced SH groups for RI folding and its activity in *E. coli *cells. To manipulate RI folding we used the classical *in vivo *approach, which is based on supplementation of the cultivation medium with low molecular weight SH group acting materials.

So far, to our knowlege, all cases targeting on the improvement of the folding of recombinant proteins with folding aiding medium additives were performed with the aim to improve disulfide bond formation. Partially SH-group modifying agents were applied in combination with other stabilising agents. For example reduced/oxidized glutathione (GSH/GSSG) and arginine can easily penetrate the outer membrane and act in the periplasmic space on the folding of disulfide bond containing recombinant proteins [10](for review see [11]). Analogically, but more sophisticated *in vivo *folding approaches in the periplasmic space were based on the utilization of low molecular additives in tandem with co-secreted chaperones [12], or overexpression of the prokaryotic disulfide oxidoreductase DsbA [13], or disulfide isomerases, such as DsbC or eukaryotic protein disulfide isomerase (PDI) [14].

In difference to the periplasmic space, the cytoplasm is considered to be reduced and thus should be the preferred compartment for expressing a protein which contrains reduced cysteins. Externally added components can also affect the cytoplasm, as was reported by Gill et al. [15]. Folding and activtiy of chloramphenicol acetyltransferase (CAT) in the cytoplasmic space was altered due the presence of dithiothreitol (DTT) in the cultivation medium, i.e. DTT is also applicable to influence the redox state in the cytoplasm and consequently may be applicable for folding control in the cytoplasm.

By taking these earlier folding cases into account, and also considering the RI structural aspects, our intention was to generate and control a favourable redox situation for RI folding in the cytoplasmic and periplasmic compartments by applying reduced glutathione which is acting in the periplasmic space, and respectively, membrane permeable DTT which is acting in both compartments. This is highly interesting, as so far all studies only improved the redox conditions during periplasmic production by using the above mentioned methodologies. Also, all approaches aimed for disulfide production rather than keeping cysteins in a reduced state. Here, to our knowledge, for the first time we show the efficiency of this approach also for the production of proteins which need a strongly reducing environment. Surprisingly this approach worked well not only for periplasmic production, but also was necessary and working for cytoplasmic production. Aside from showing the feasibility of this approach at the example of an RI we go one step further and demonstrate that this approach is well suited also as a production strategy in typical fed-batch processes.

## Results

The aim of the study was to produce correctly folded RNase Inhibitor (RI) in *E. coli*. We aimed to test whether it would be possible to produce RI as an authentic active protein (without any fusion) in the periplasmic or cytoplasmic compartments. We presumed that it would be possible to control the conditions in the periplasmic and cytoplasmic space by either process parameters or chemical additives.

### Library screening in 96 well plates

In order to evaluate the best conditions for secretion of RI to the periplasmic space of *E. coli*, the RI gene was cloned by Gateway cloning into a periplasmic expression library which was earlier described [16]. This library contains a set of 36 different ColE1-derived plasmid vectors being a full factorial combination of three varying parameters: each three different IPTG inducible promoters and ribosome binding sites of different strength, and four well known signal peptides for translocation of the product protein into the peripasmic space. The RI gene harboring set of plasmids was transformed to the *E. coli *K-12 strain RV308 which additionally contained the plasmid pLT1 based reporter system for the monitoring of periplasmic folding stress by a *degP *promoter controlled luciferase cassette [16]. The σ^24 ^dependent *degP *promoter is induced by periplasmic folding stress, i.e. if the protein of interest would aggregate during the accumulation in the periplasmic space.

**Figure 1:**
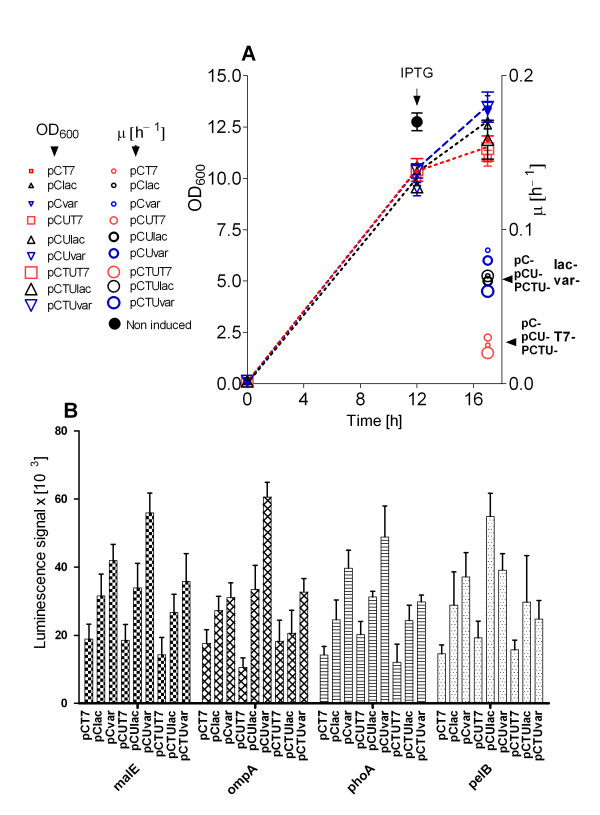
**Growth with EnBase in MWPs of a library of 36 constructs with periplasmic RI production and luciferase activity of the different clones. A:** OD_600_'s are shown for all cultures of the vector library. Label sizes represent promoter strength [from weakest (pC, smallest labels) to strongest (pCTU, largest labels)]. Label shapes and colours represent ribosome binding strengths: T7 (red squares), lac (black triangle), var (blue triangle). Sizes and colours of the specific growth rate labels (circles) also indicate promoter and ribosome binding site strengths. **B**: The periplasmic aggregation signal measured as luminescence in samples of the RI expression library 5 h after induction. The data represent three independent cultivation experiments of the whole library.

As in our earlier study the initial screening experiments were performed in 96-well plates. For obtaining (i) well controlled conditions, (ii) enough cell material, i.e. high cell densities, and (iii) additionally applying strategies which would be applicable in the fermentation scale later, again, we were applying the EnBase cultivation technique in MWPs with pure mineral salt medium and starch-derived glucose as carbon source (cf. [8,17]). The EnBase-gel containing MWPs were directly inoculated with glycerol stocks of all 36 strains. Periplasmic RI synthesis was induced after 12 hours of cultivation by IPTG (Figure 1), the temperature was decreased to 22°C and 5 h later the cultures were harvested.

All constructs showed a good accumulation of RI. Remarkably, a 20-30% higher yield of RI was detected in the strains carrying vectors with the stronger promoters (pCU, pCTU) in combination with the strongest ribosome binding site (T7)(gels images not shown). However, unexpextedly, all samples showed a very low activity, independently on the expression strength or signal peptide (gels images and graphs not shown). These results would suggest that RI is expressed, but accumulated in inclusion bodies in all cases. However, intrerestingly this was not reflected by the luminescence signals in the different strains (cf. Figure 1). High luminescence signals were not connected to high RI accumulation levels (i.e. aggregated RI), but opposite - higher luminescence values were generated in the strains with the weaker promoters (pCU, pC) in combination with the weaker ribosome binding sites (var, lac). Therefore we consider for RI the periplasmic folding stress reporter system is not applicable to identify conditions for soluble periplasmic RI accumulation. This is clearly different to the earlier used cytoplasmic monitoring system [8] and to the results by Kraft et al. [16] with the scFv-miniantibody-phosphatase fusion and 11-β-hydroxysteroid dehydrogenase type 2 which were expressed in the same periplasmic expression library.

It may be worth to mention that the culture growth after induction of RI is dependent on the strength of product synthesis. The cell growth after RI induction of the clones harboring vectors with the higher synthesis rate stipulating elements, i.e. pCU, PCTU and T7, was strongly inhibited compared to cultures harboring vectors with weaker expression elements (pC, pCU, lac, var) which continued to grow (Figure 1).

### Addition of reducing compounds

In summary, the first set of experiments showed that RI is well produced with some of the constructs, but that the product is neither active nor soluble. One major reason for the aggregation during recombinant production in *E. coli *could be the inability of the expression host to stably maintain the SH groups of the target RI. Therefore, we decided to supplement the cultivation medium with low molecular weight SH group stabilizing agents, such as DTT and reduced glutathione (GSH). Both are known to easily access the periplasmic space. Additionally, in parallel, target RI sensitivity to oxidation during the periplasmic accumulation was tested with and without reducing agents in an isogenic *dsbA *knockout mutant of the RV308 strain.

All these second round experiments were performed in shake flasks, with only a part of the set of plasmid constructs. The study was continued with the clones which during the screening in 96-well microwell plates resulted in the highest (pCUvar with *malE*, *ompA*, *phoA *signal peptides; and the pCUlac promoter with the *pelB *signal peptide - referred as the "first group") and the lowest luminescence levels (pCTUT7- *malE*, *ompA*, *phoA*, *pelB *- referred as the "second group"), respectively.

**Figure 2:**
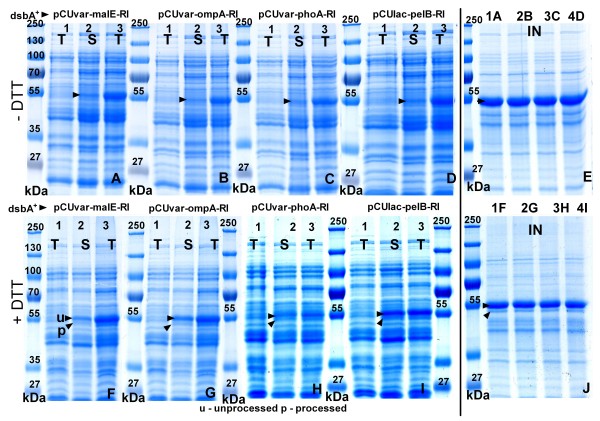
**SDS-PAGE images of total (T), soluble (S), or insoluble (IN) protein fractions of samples from shake flask cultures of *E. coli *RV308 *dsbA^+ ^*with the following periplasmic constructs: pCUvar-malE-RI (A, F), pCUvar-ompA-RI (B, G), pCUvar-phoA-RI (C, H) and pCUlac-*pelB*-RI (D, I) 4 h after induction**. Gels represent protein fractions after RI production without DTT (A-E) or with 12 mM DTT (F-J). Gels E and J represent insoluble protein fractions of the constructs without DTT (E) or with 12 mM DTT (J). Numbered lanes: 1 - total protein fraction 10 min before induction, 2 and 3 - soluble and total protein fractions 4 h after induction. Numbered lanes in gels E and J represent the insoluble fractions with the plasmids pCUvar-malE-RI (1A, 1F), pCUvar-*ompA*-RI (2B, 2G), pCUvar-*phoA*-RI (3C, 3H) and pCUlac-*pelB*-RI (4D, 4I). Protein size marker: PageRuler™ Protein Ladder Plus (Fermentas). For growth conditions see Materials and Methods. The amounts of applied protein are normalised.

**Figure 3:**
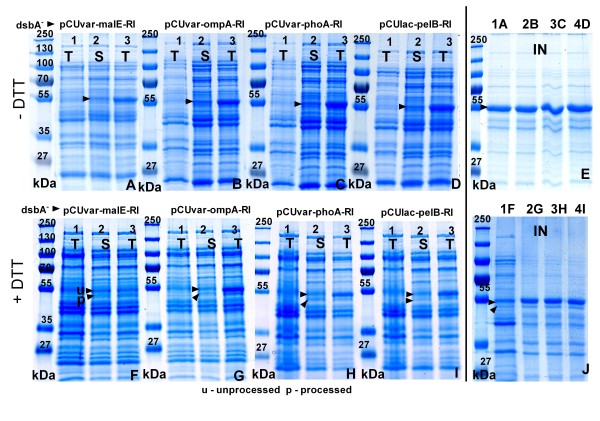
**SDS-PAGE images of total cell extracts (T), soluble (S), or insoluble (IN) protein fractions from periplasmic production constructs of *E. coli *RV308 *dsbA*^-^**. For vectors and conditions see Fig. 2.

Cultivations were performed in glucose-MSM as described in the Material and Methods part. At the time of induction the cultivation medium was supplemented with the reducing agents DTT or GSH, respectively, in different concentrations. The results showed no effect on RI activity or improvement of RI accumulation in the soluble fraction in the cultures with 20 or 50 mM of GSH (graphs not shown). Only DTT resulted in a significant improvement of RI accumulation in the soluble fraction and also in an increased RI activity in both, *dsbA^- ^*and *dsbA*^+^, strains (Figures 2, 3). SDS-PAGE analysis of protein fractions from cultures with addition of DTT revealed an increasing intensity of 2 bands; their molecular sizes were corresponding to RI with a signal sequence (53 kDa) and without (50 kDa). The identity of the processed RI (50 kDa protein) was confirmed by N-terminal sequencing of 6 amino acids.

SDS-PAGE analysis showed that the highest amounts of unprocessed (app. 28 mg gCDW^-1^) and processed RI (app. 12 mg gCDW^-1^) were obtained in the RV308 *dsbA^+ ^*strain in the soluble protein fraction when the cultivation was performed with the pCUlac-*pelB*-RI vector with 12 and 18 mM of DTT. Under these conditions the yield of processed and even of unprocessed soluble RI protein was improved by 2 to 2.5-fold compared to the control without DTT (Figure 2). Different amounts of DTT did not affect the total amount of accumulated RI in the *dsbA^+ ^*strain, but both, DTT and GSH, had a highly negative impact on the growth and RI yield in the *dsbA^- ^*strain.

**Figure 4:**
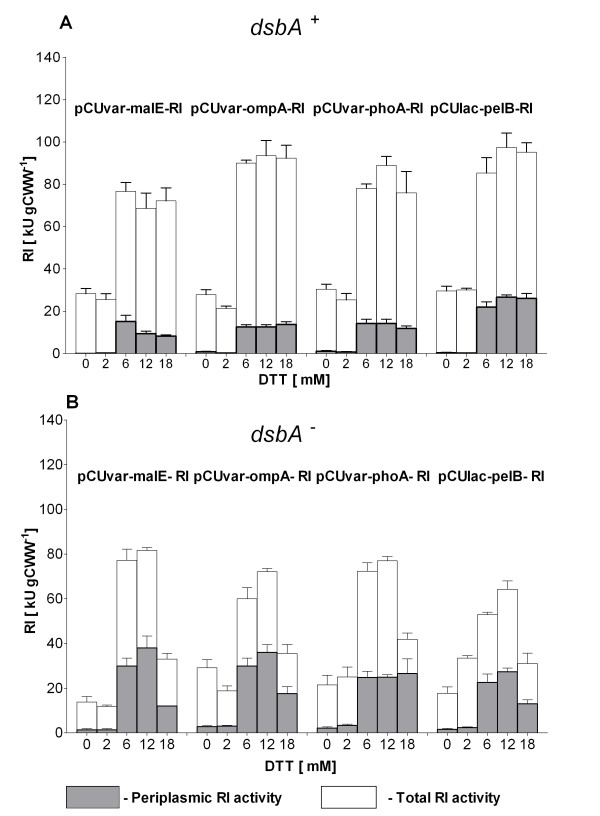
**RI protein activities in total soluble and periplasmic fractions after 4 h of batch RI production in different *E. coli *RV308 *dsbA^+ ^*(A) and *dsbA^- ^*(B) constructs at 22°C with 0, 2, 6, 12 or 18 mM of DTT**. Culture conditions: glucose-MSM medium, induction with 0.2 mM IPTG, preinduction temperature 37°C, shift to the respective temperature at the time of induction.

After periplasmic production without DTT in the medium, RI activity was detected only in the *dsbA*^- ^strains (Figure 4). Depending on the DTT concentration in the medium, total (soluble and insoluble) RI amounts in the *dsbA^- ^*strain constructs were 2 to 4-fold lower compared to the controls without reducing agents (Figure 3). The negative effect of DTT on the accumulation of RI resulted also in a lower activity; cultures with 18 mM of DTT showed a 2 to 3-fold lower activity compared to the cultures with 12 mM DTT (Figure 4).

Despite their bad DTT tolerance, the yield of processed RI was improved in all *dsbA^- ^*constructs when a lower DTT concentration was used (6 and 12 mM). In the *dsbA^- ^*strains the highest yields of processed and soluble RI (app. 7 mg gCDW^-1^) were obtained with the pCUvar-*malE *construct. In the other constructs the yield was 10 to 20% lower (Figure 2). In the *dsbA^- ^*strains the yield of unprocessed RI was very similar for all constructs, but indeed 2 to 4-fold lower compared to the *dsbA*^+ ^strain. Also, the amounts of processed RI were highest in the *dsbA*^+ ^strain (30% higher than in the *dsbA*^- ^strain) (Figures 2, 3).

The analysis of the insoluble protein fraction showed that after RI production with 6 to 12 mM DTT processed RI appeared in the insoluble protein fractions of both, *dsbA^+ ^*and *dsbA*^- ^strains (Figures 2, 3).

Despite the significantly higher amounts of soluble RI achieved in the *dsbA^+ ^*strain, RI activities in total protein fractions of the *dsbA*^- ^strain were just 10% lower. Interestingly, if the analysis was performed with the periplasmic fractions only, the *dsbA*^- ^strain constructs showed even a 1.5 to 2-fold higher RI activity in comparison to the *dsbA^+ ^*strain (6 and 12 mM DTT, Figure 4).

The results of these experiments indicate that 12 mM of DTT is optimal for RI production in *dsbA^- ^*and *dsbA^+ ^*strains. In contrast, GSH was not efficient for RI periplasmic accumulation. Thus it was not used in the further experiments.

Analogous experiments with the second construct group with the strongest promoters and ribosome binding sites (pCTUT7 constructs) also showed that the addition of 12 mM DTT to the medium was optimal for the RI production. However, compared to the first constructs, this second group resulted in a 4 to 5-fold lower yield of active and soluble RI. Remarkably, no RI activity was found in the periplasmic protein fractions of the *dsbA^+ ^*and *dsbA*^- ^strains of this second group of constructs (graphs not shown).

### Cytoplasmic RI production with DTT

Based on the results with the periplasmic expression vectors, which (i) clearly showed a positive effect of DTT not only on the processed form of RI but also for the unprocessed, i.e. signal peptide contaning form, we expected that the same approach also should improve the yield of cytoplasmically expressed RI. To evaluate this we selected the *E. coli *RV308 pCUlac-His6-RI construct which was earlier constructed (see [8]). In this construct a 6× histidine tag is fused to the N-terminus of RI. Otherwise this cytoplasmic construct is simlar to the periplasmic production construct pCUlac-*pelB*-RI.

**Figure 5:**
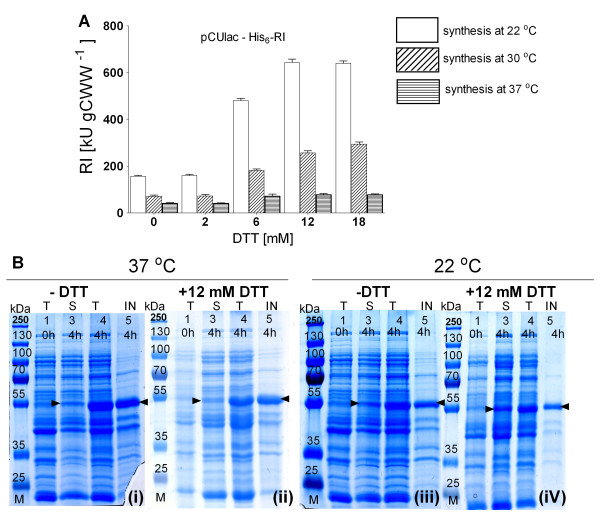
**RI protein activities in total soluble fractions (A) and SDS-PAGE images of normalised total (T), soluble (S) and insoluble (IN) cell extracts (B) after 4 h of batch cytoplasmic RI production in shake flask batch cultures in *E. coli *RV308 pCUlac-His_6_-RI at 22°C with 0, 2, 6, 12 or 18 mM of DTT**. Lanes in (**B**): 1 - total protein fraction 10 min before induction, lanes 2 to 4 - soluble, total and insoluble protein fractions 4 h after induction. Gels represent protein fractions after 4 h of RI production without DTT at 37°C (gel 1) or 22°C (gel 2), or with 12 mM DTT at 37°C (gel 3) or 22°C (gel 4).

The experiments for cytoplasmic RI production were performed analogously to the periplasmic expression with addition of different amounts of DTT at the time of induction. Here, clearly the RI activtiy was affected by the synthesis temperature and the DTT concentration (Figure 5). In more detail, DTT did not affect the yield of soluble RI if the production was performed at 37°C with all tested concentrations of DTT. Furthermore, 2 mM of DTT did not affect protein accumulation and activity of RI in the soluble fraction, independently from the synthesis temperature (Figure 5).

An obvious positive effect of DTT on the accumulation and activity of RI in the soluble fraction was detected if the production was performed at 30 or 22°C with at least 6 mM DTT. RI activities were increased by app. 30% compared to the controls without DTT. A slight improvement of the RI activity was also observed even after production at 37°C with 12 and 18 mM of DTT (Figure 5). Indeed, the best results for cytoplasmic production were achieved after synthesis at 22°C with 12 or 18 mM of DTT in the medium, corresponding to ≈36 mg gCDW^-1 ^and ≈620 kU gCWW^-1 ^respectively (Figure 5).

Interestingly, if the yields between cytoplasmic and periplasmic cultures are compared, it is remarkable that the amounts of soluble RI were highly similar to cytoplasmic yields in the best periplasmic production constructs. Surprisingly, the RI actvity was even 3-fold higher with the cytoplasmic systems under comparable conditions.

### RI production in fed-batch shake flasks with EnBase

After the optimal conditions for periplasmic and cytoplasmic RI production were defined, the next challenge was to test the effect of DTT on product accumulation and folding in the bioreactor under fed-batch conditions. Prior to the fed-batch bioreactor experiments the RI periplasmic and cytoplasmic production with 12 mM DTT in the medium were evaluated under fed-batch conditions in shake flasks by applying the EnBase technology. The experiments were performed with the following constructs: (i) for the periplasmic production the highest amount of processed and active RI yielding constructs: RV308 *dsbA^+ ^*pCUlac-*pelB *and RV308 *dsbA^- ^*pCUvar-*malE*, (ii) for cytoplasmic production RV308 pCUlac-His6-RI.

**Figure 6:**
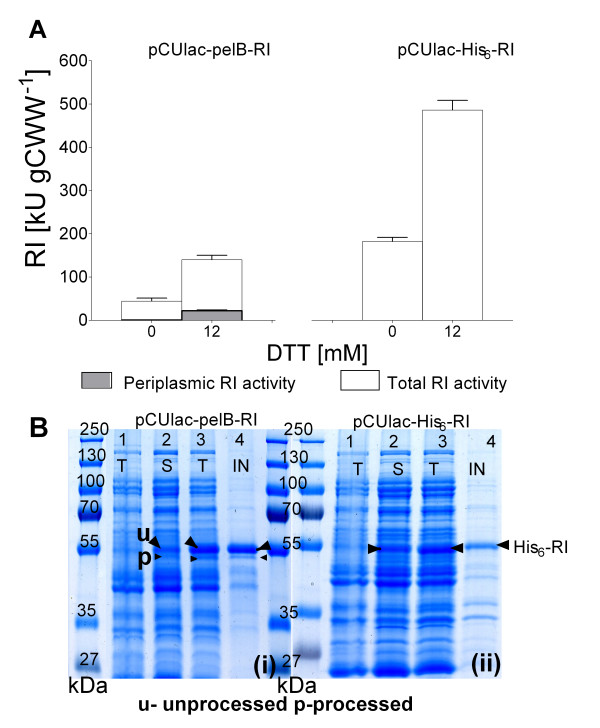
**RI protein activities in total soluble and periplasmic fractions [in kU (gCWW)^-1^] of *E. coli *RV308 pCUlac-pelB-RI and *E. coli *RV308 pCUlac-His_6_-RI after EnBase cultivation in shake flasks with RI induction for 4 h at 22°C without or with 12 mM DTT (A), and SDS-PAGE images of total (T), soluble (S) and insoluble (IN) protein extracts (normalised to equal amounts protein) (B)**. Left gel: *E. coli *RV308 dsbA^+ ^pCUlac-*pelB*-RI (periplasmic expression construct); right gel: *E. coli *RV308 pCUlac-His_6_-RI (cytoplasmic production construct). Lanes: 1 - total protein fraction 10 min before induction, 2 to 4 - soluble, total, and insoluble protein fractions 4 h after induction with 12 mM of DTT. Protein size marker: PageRuler™ Protein Ladder Plus (Fermentas). Data originate from three experiments.

All cultures performed with the *dsbA^+ ^*strain showed a 2.6-fold improved RI production under the substrate limited condition, in both, the periplasmic and cytoplasmic constructs. However, in contrast to the earlier performed batch cultures, fed-batch production resulted in a 15 to 20% lower amount of active RI per cell unit (Figure 6). Under substrate limited conditions the *dsbA^- ^*strain grew very poorly and no product could be detected. Therefore, aside from the cytoplasmic constructs, only the periplasmic expression construct RV308(*dsbA*^+^) pCUvar-*malE *was used in the following bioreactor experiments.

### RI production in bioreactors

Finally, batch and fed-batch periplasmic and cytoplasmic production of RI was performed in a stirred bioreactor.

**Figure 7:**
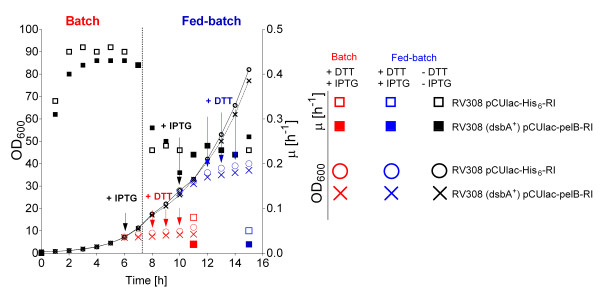
**Growth curves of *E. coli *RV308 pCUlac-*pelB*-RI (periplasmic expression construct) and *E. coli *RV308 pCUlac-His_6_-RI (cytoplasmic production construct) without (control) and with RI production in a batch and a fed-batch process with exponential glucose feeding in a 10 L bioreactor**. For conditions see Material and Methods. Black symbols: control culture without induction; red symbols: batch bioreactor cultivation, blue symbols: fed-batch bioreactor cultivation.

In batch cultivations induction was performed at an OD_600 _of 7 (μ≈0.45 h^-1^). In all fed-batch processes RI production was induced during the glucose-limited growth phase with exponential feeding at an OD_600 _of app. 28 (μ≈0.22 h^-1^). After induction the feeding rate was further increased according to the predetermined feed function with the same μ_set _as before induction. Also DTT was added at the time of induction as in the earlier experiments and concomitantly the temperatre was decreased from 37 to 22°C (Figure 7).

**Figure 8:**
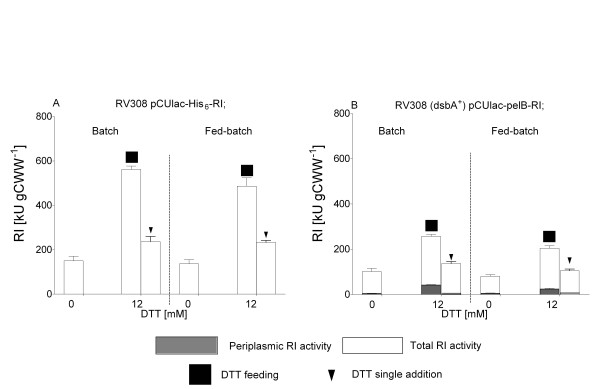
**The amount of reduced DTT during batch shake flask and fed-batch bioreactor processes with microarerobic production mode, was measured by using Measure-iTTM Kit (Invitrogen)**. The samples for reduced DTT evaluation were taken every synthesis hour starting with DTT addition moment. The data is derived from three assays.

DTT is inactivated during bioreactor cultivation with a faster rate compared to shake flask cultures where the DTT oxidation rate was very low (Figure 8). After 4 hours of RI production in shake flasks only 5 to 10% of DTT was oxidized. In contrast more than 50% of DTT were oxidized during bioreactor cultivation already after 3 h. Therefore different approaches were tested and compared to keep the reduced state in the culture: (i) Single pulse addition of DTT to a final concentration of 12 mM at the induction point, (ii) single pulse addition of DTT to a final concentration of 12 mM 2 h after RI induction, and (iii) repeated (3 times) addition of DTT, starting at 2 h after induction (Figure 7).

**Figure 9:**
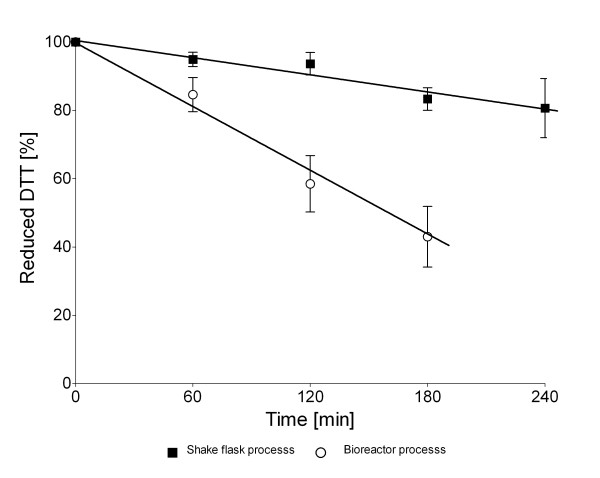
**RI activities in the total soluble protein fraction (white bars) and in the periplasmic fraction (grey bars) of *E. coli *of RV308 pCUlac-*pelB*-RI and RV308 pCUlac-His_6_-RI from samples of batch and fed-batch bioreactor cultivations without and with addition of DTT**. Data derive from three activity assays.

Additionally, in order to prevent rapid DTT oxidization in all processes at the DTT addition point, the air flow was reduced from 30 L min^-1 ^to 2 to 3 L min^-1 ^to maintain the oxygen concentration in the medium close to zero. The reduction of the air flow was a necessary condition for accumulation of active product. Only at an reduced air flow rate the additon of DTT provoked a clear positive effect with highest yields, and interestingly this worked for both, the periplasmic and the cytoplasmic expression systems. However, despite a 30-fold higher volumetric productivity in the fed-batch bioreactor processes compared to the analogous batch processes, the periplasmic and cytoplasmic RI yields per cell remained 10 to 15% lower in the fed-batch cultivations with the same DTT addition mode (Figure 9).

Protein activity analysis in the crude extracts of samples from the fed-batch cultures revealed - different from the shake flasks - that RI activity was only improved by 1.4-fold by single DTT addition, but 2 to 3-fold when the repeated DTT addition approach was applied (Figure 9). This is not surprising, because even the minimum air flow, maintaining 0% of oxygen concentration in the medium, resulted in 60% of DTT oxidation during the whole bioreactor process (Figure 8).

## Discussion

In this work, for the first time, low molecular weight SH group modifying agents were utilized for periplasmic and cytoplasmic folding improvement of a cysteine-rich LRR model protein with exclusively reduced cysteins, a recombinant ribonuclease inhibitor (RI). We demonstrated the effect of SH group modifying agents on periplasmic and cytoplasmic activities of RI and its soluble accumulation. We also showed that the reason for the non-successful approaches for expression of active RI in *E. coli *is its incapability to create an optimal redox environment for RI folding even in the cytoplasm. In addition, obviously RI folding *in vivo *does not just depend on the redox conditions. Only the combination of lower translation rate, low post-induction temperature and strongly reducing conditions resulted in a reasonable yield of RI and these conditions could be applied for periplasmic as well as for cytoplasmic RI production. Furthermore, on the basis of these principles, which were evaluated in small-scale cultures, production of RI was also successful during fed-batch cultivation in a stirred tank bioreactor.

### Screening of the best constructs for soluble production of RI

In the first part of the work, for evaluation of significance of keeping reduced conditions, RI folding was compared by expressing RI in the periplasmic compartment of the *E. coli *K-12 strain RV308 and its isogenic *dsbA^- ^*mutant. This approach was based on the well known fact that the redox status in the periplasm could be easily controlled by the addition of SH group stabilizing agents, such as DTT and GSH, to the cultivation medium. However, previous approaches mostly aimed for optimising the redox conditions for disulfide bond isomerisation (e.g. [12-16]). As a first step, however, we tested whether different combinations of promoter, ribosome binding site and signal sequences stipulate soluble RI accumulation in periplasmic space as a kind of initial standard conditions. Therefore we applied a previously published plasmid library for periplasmic production together with a periplasmic folding reporter system [16]. The periplasmic RI folding in all production constructs was evaluated by using the experimental set up published recently by Šiurkus et al. [8], combining fed-batch RI production in MWPs with luminescence based high throughput screening. Interestingly, and in difference to the earlier published results with the screening for soluble human epidermal growth factor receptor and human 11β-hydroxysteroid dehydrogenase type 2, in case of RI the screening results did not match with the soluble product amount in the periplasmic space. In our case the weaker expression elements harbouring periplasmic production constructs gave higher luminescence signals compared to the strong pCTUT7 constructs. We suggest that in case of very poor product solubility, and thus high luminescence signals already in the weaker expression vectors, lower luminescence in the stronger expression vectors is a result of lower luciferase production by an overloaded cellular production machinery. Accordingly, the strongest growth inhibitory effect was observed for the pCTUT7 constructs in which the expression of luciferase was lowest. Weaker expression stipulating constructs (promoters pC-, pCU-, RBS: -lac, -var) resulted in high luminescence signals during periplasmic RI production because a part of the product was found in periplasmic inclusion bodies on one side, but on the other side the weaker target protein expression in these vectors did not consume all cellular recourses needed for luciferase production and cellular growth. Thus in summary, luciferase signals in combination with the periplasmic library have to be evaluated with caution.

As the initial screening results provided no clear answer on the preferable constructs, both, a strong and a weaker expression construct were selected for the further studies. As the previous results [8] indicated aggregation in the cytoplasm as a major problem, the following work was focussed on the control of the redox conditions. This was parallel approached by using a *dsbA^- ^*mutant to remove of the strong oxidising activity of DsbA, and in parallel applying reducing agents. Interestingly, in our case GSH was not active, neither in the *dsbA^+ ^*nor in the *dsbA^- ^*mutants. However, in contrast DTT worked well. More favourable conditions for RI folding in the periplasmic space were created in the *dsbA^- ^*mutant, but this mutant seemed also to be highly sensitive to DTT and could not be productive in the presence of higher concentrations than 6 mM DTT. By considering all results of the separate analysis of the amounts of processed (i.e. without signal peptide) and non-processed forms of RI (i.e. with signal peptide), we conclude that RI accumulation in the periplasmic space was improved due to the primary effect of DTT on the cytoplasm, where it avoided aggregation of the protein, even in the case of cytoplasmic RI expression vectors.

The significance of the synthesis rate of RI for its periplasmic accumulation and folding was clearly demonstrated from RI periplasmic production experiments with DTT in the strong and weak expression stipulating constructs groups. The results showed that DTT was highly effective only in weaker expression rate stipulating constructs, the balance between the synthesis and the folding rate is important for obtaining soluble product. In contrast DTT was not effective for RI folding in the strong expression elements harbouring constructs in which obviously the RI synthesis rates were too high.

The leader peptide had a lower impact on the accumulation of processed RI. For further studies the *pelB *leader peptide was selected as the most suitable for RI periplasmic accumulation in dsbA^+ ^strain. However, also the *ompA*, *malE *and *phoA *leader peptides stipulated the periplasmic accumulation of RI in the presence of DTT.

Our results are in good agreement with other periplasmic production cases. For example the significance of synthesis rate on periplasmic accumulation and aggregation of recombinant penicillin G acylase was also demonstrated by Sriubolmas et al. [18]. The authors showed that the cytoplasmic and periplasmic aggregation of penicillin G acylase depend on the synthesis rate, which was altered by varying the amounts of IPTG. In addition, RI accumulation patterns, obtained after RI synthesis with DTT, represented by premature and processed RI forms in the insoluble and soluble protein fractions, are typical for periplasmic production. Similar pattern were reported by Sriubolmas et al. [18] for penicillin G acylase and by Bowden et al. [19] for β-lactamase. In both cases a mixture of precursor polypeptides with signal peptides and processed periplasmic proenzyme forms was detected.

Finally, another interesting optimisation case may be mentioned, involving the same vector library and the periplasmic misfolding reporter. Soluble periplasmic accumulation of human 11β-hydroxysteroid dehydrogenase type 2 and scFv-miniantibody phosphatase was highly dependent on the leader peptide but not on the expression rate regulating genetic elements. Indeed, lower periplasmic aggregation levels and more efficient export to the periplasmic space were observed for the constructs with the weaker pCU promoter and the *lac *ribosome binding site, compared to the strong expression pCUT7 vectors. [16]

### The effect of DTT on the RI cytoplasmic folding

The RI activity was clearly dependent on the DTT concentration in the medium. Additionally, besides DTT, cytoplasmic folding of RI strongly depended on the production temperature as a synthesis rate and folding regulating factor. Even when RI production was carried from a weaker promoter and ribosome binding site, the lower production temperature stipulated a better RI folding. Compared to best periplasmic production results, the cytoplasmic production construct gave 3 to 4-fold higher total RI activity, although just 30% less premature RI was produced in the best periplasmic production construct. We suggest that the non-processed N-terminal signal sequence, which is 3-fold longer compared to the 6× His-tag can negatively affect the ability of RI to interact with Rnase E. A negative effect of an N-terminal tag on the activity of RI was also observed after RI production as a fusion with MBP where the RI specific activity was about 12-fold reduced (Šiurkus unpublished data).

In our opinion, the RI solubility was improved due to the complex DTT effect on (i) RI SH groups and (ii) reduced expression rate stipulated by DTT toxicity, genetic elements and lower production temperature. Without doubt DTT could also have a negative impact on the overall target protein yield due to the highly induced stress related proteins as reported by Han et al. [20]. Gill et al. [15] reported increased protease activities and heat shock protein synthesis in *E. coli *JM105 and RR1 strains after recombinant CAT production in a bioreactor due to the presence of relatively low amounts of DTT in the medium. Surprisingly our strain demonstrated a comparably high tolerance to DTT. It was still very productive in the medium containing a total DTT concentration of close to 20 mM which according to Missiakas et al. [21] should be a lethal for *E. coli*.

Interestingly, DTT served as an SH group modifier *in vivo *not just in the periplasmic space but also in the cytoplasm. It was obvious from the cytoplasmic production results that the redox environment in the *E. coli *cytosol is not optimal for a target protein with a high content of reduced cysteins. The *E. coli *cytoplasmic environment is in general reduced, but oxidative damage occurs when cells enter the stationary phase and starvation [22,23]. That would possibly lead to target protein SH group oxidation.

### RI fed-batch production in shake flasks

Enbase experiments provided valuable information for the process development in the bioreactor. The *dsbA^- ^*strain turned out to be inable to maintain its productivity under substrate limited feed conditions with DTT and thus would not be favorable for futher bioprocess development. In contrast the cytoplasmic and periplasmic fed-batch production patterns in the constructs with the *dsbA^+ ^*strain with 12 mM of DTT were similar to the batch shake flasks, showing that the substrate limited cultivation mode has no negative effect on the host productivity and protein folding in our case. Thus Enbase clearly helped to save time and labor in hte process develpment process.

### Bioreactor processes

The bioreactor experiments showed that highly aerated cultivation medium could be a very oxidative environment. That should be considered when compounds which are sensitive to oxidation are used for protein folding, recombinant synthesis induction and/or plasmid stabilization. Microaerobic or fully anaerobic production strategies would preserve more oxidation sensitive chemicals. On the over hand respiration is a key factor for recombinant productivity. Anaerobic conditions generally result in poor growth and are often considered as unfavourable for recombinant protein production, by limited energy production, acidification of the cytoplasm by organic acids and the large synthetic requirements which are needed to establish the anaerobic responses [17,24,25]. Accordingly, we did not succeed to produce RI when the air flow was downregulated at the induction point, however, we could solve the problem by later addition of DTT and concomittant reduction of the airflow.

In our case DTT oxidation in the bioreactor was the main concern during development of the bioreactor process. We succeeded to prevent rapid DTT oxidation, which was obvious if the DO was maintained at approximately 30%, by strongly reducing the aeration. Thus we created conditions which are usually present in shake flasks [17]. After medium supplementation with DTT the air flow was not completely switched off, but down-regulated in order to maintain the actual oxygen concentration close to 0%.

This down-regulation of the air flow rate had a drastic effect on the cell productivity when it was performed at the same time with DTT addition and RI synthesis induction. In this case no RI production was found in periplasmic and cytoplasmic constructs. In our opinion the combination of temperature reduction, induction and DTT addition stipulated a huge metabolic burden as earlier defined by Glick [26]. In order to reduce the stress, we decided to induce recombinant production separately from medium supplementation with DTT and down-regulation of the air flow. Thus only the temperature shift was performed at the time of induction, but the reducing agent was added only 2 hours after induction when the RI production reached its maximum, and at the same time the air flow was reduced.

Furthermore, unexpectedly, after single medium supplementation with DTT much lower RI activity per cell unit was detected in the bioreactor production process compared to the shake flasks. However, the total RI production level per cell unit in the bioreactor was similar to the shake flask results. We suspected that the decreased RI activity in the bioreactor could be related to the partial oxidation of the DTT even at the very low air flow rate, as confirmed by the analysis of the DTT oxidation rate. Whereas during RI production only 10% of the DTT was oxidized in shake flasks, 60% was found to be oxidised in the bioreactor even at microaerobic conditions. To counteract this we tested whether repeated DTT addition would improve the yield of active RI. As expected, repeated DTT addition strongly enhanced RI folding during batch and fed-batch processes. In this case the amount of active RI per cell unit in the bioreactor processes was similar to the activities which were detected in samples from shake flasks. The effective RI production with DTT under batch and fed-batch conditions shows that the *in vivo *approach for RI folding is reproducible, independently from the cultivation mode and cell densities.

## Conclusions

In this study we demonstrate the successful production of active RI by periplasmic and cytoplasmic approaches based on the artificial control of the redox conditions and the expression rate via external manipulations with medium components and cultivation parameters. The folding approach presented here could be very useful for recombinant protein production not just distinguished by reduced SH groups but also for disulfide bond containing proteins. In our opinion the combination of oxidised/reduced DTT in tandem with the cellular oxidation/reduction machinery and cultivation parameters could enhance oxido-(re)shuffling needed for correct formation disulfide bonds in the cytoplasm. Our folding approach could be applied as an alternative for protein synthesis in the periplasmic compartment where the main synthesis bottleneck is protein transfer across the periplasmic membrane.

## Methods

### Vector library preparation

A previously described periplasmic expression library [16], containing 36 periplasmic expression vectors, was kindly provided from Hans-Knöll-Institute, Jena, Germany. The RI encoding gene was inserted into periplasmic expression vectors via site specific recombination reaction based on the Gateway^® ^cloning technology (Invitrogen) as described by [8].

#### Preparation of target protein expression platforms

The expression strain *E. coli *K-12 RV308 (ATCC 31608) was first transformed by using the calcium temperature shock method with the periplasmic folding stress reporter plasmid plt1 previously described by Kraft et. al. [27], carrying a resistance for ampicillin, and plated on LB agar with ampicillin (100 μg mL^-1^). RV308 plt1 was co-transformed with the library of 36 the RI gene containing periplasmic expression vectors. The transformants were plated on LB agar containing ampicillin (100 μg mL^-1^) and chloramphenicol (30 μg mL^-1^). The cell stock was produced after 8 h of recombinant strain cultivation in 10 mL of LB medium in 100 mL shake flasks at 37°C and 220 rpm. All culture suspensions with OD_600 _of 4 ± 0.2 were mixed with an equal volume of sterile 50% glycerol solution to achieve a final glycerol concentration of 25%. The glycerol culture suspensions were aliquoted into sterile PCR strips and stored at -70°C. For RI cytoplasmic expression experiments the previously described RI expression construct RV308/pCUlac-His_6_-RI [8] was used.

### Engineering of E. coli RV308 dsbA^- ^strain

The *dsbA *gene in RV308 was in inactivated by P1 transduction. E. coli JW3832 from Keio collection was used as the donor for the *ΔdsbA::kan *marker. The RV308 clones harboring *ΔdsbA::kan *were selected after cultivation on solid LB medium containing kanamycin antibiotics. In addition, the mutation in selected clones was confirmed by PCR analysis, with the following forward and reverse primers: 5'-aagatttggctggcgctggct-3' and 5' - tcggacagatatttcactgtatca - 3. The strains - RV308 *dsbA^+ ^*and JW3832 *ΔdsbA::kan *were used as controls in the PCR analysis.

### Cultivation media

Transformations and plasmid propagations were performed on solid and liquid LB medium containing Bacto-Tryptone (10 g L^-1^), Bacto-yeast extract (5 g L^-1^), NaCl (10 g L^-1^), 15 g L^-1 ^bacto agar (if solid medium) and the required antibiotics. Fed-batch and batch cultivations were performed in glucose-based mineral salt medium (MSM) with the following composition (per litre): Na_2_SO_4 _2 g, (NH_4_)_2_SO_4 _2.68 g, NH_4_Cl 0.5 g, KHPO_4 _14.6 g, NaH_2_PO_4_×H_2_O 3.6 g, (NH_4_)_2_-H-citrate 1.0 g, and glucose 2.5 to 15 g. NaOH (40%) was used to adjust pH to 7.0 prior heat sterilisation. Additionally, before cultivation on the mineral salt medium the following sterile solutions were added: 3 mL L^-1 ^of (1 M) MgSO_4 _and 2 mL L^-1 ^of trace element solution with the following composition (per litre): CaCl_2_×2H_2_O 0.5 g, ZnSO_4_×7H_2_O 0.18 g, MnSO_4_×H_2_O 0.1 g, Na_2_-EDTA 20.1 g, FeCl_3_×6H_2_O 16.7 g, CuSO_4 _× 5H_2_O 0.16 g, CoCl_2 _× 6H_2_O 0.18 g; and thiamine hydrochloride (0.1 mM), ampicilin (100 mg L^-1^) and chrolamphenicol (30 mg L^-1^). The feeding solution for fed-batch cultivations was based on fully formulated MSM with the required antibiotics and 550 g L^-1 ^of glucose.

### Fed-batch mode cultures and recombinant protein synthesis in 96 microwell plates

Agar based EnBase^® ^96 microwell plates (MWPs) were purchased from BioSilta Oy Oulu (Finland). For cultivations in MWPs 10 μl of glycerol stock cultures were transferred to cultivation wells containing 150 μl of fully formulated MSM medium with 6 AGU L^-1 ^of EnzI'm (BioSilta Oy). Periplasmic RI expression was induced after 10 hours of cultivation at 30°C at an OD_600 _of 12 ± 2 (μ ≈ 0.16 h^-1^) by addition of IPTG (0.2 mM final concentration). All microscale cultures were cultivated by intensive shaking with a Variomag^® ^Thermoshake (Inheco, Germany), shaking diameter 1.5 mm, at 30°C and 750 rpm. After induction the temperature was decreased to 22°C and the cultures were harvested 5 h after induction. The OD_600 _measurements and the luminometric assay for assaying the target protein periplasmic misfolding levels in MWP's were performed as earlier described [8].

### Batch mode cultivations in the shake flasks

The inoculums for batch protein production in shake flasks were prepared by overnight batch cultivation of the selected clones in 500 mL shake flaks with 50 ml of glucose-MSM with 10 g L^-1 ^of glucose at 37°C. For protein production 5% of the corresponding inoculum culture was transferred to fresh glucose-MSM containing the same amount of glucose at a final volume of 200 mL in 1 L baffled Erlenmeyer flasks. Cultures were grown at 37°C and 180 rpm until they reached a cell density of OD_600_= 1 ± 0.05 (μ ≈ 0.35 h^-1^) where induction was performed by addition of IPTG (final concentration 0.2 mM). DTT was added to the cultivation medium at the RI induction point as dry powder to achieve the needed concentration of 2 to18 mM, and reduced glutathione was added with a final concentration of 20 or 50 mM, respectively. The temperature was shifted at the time of induction to 22°C and the cultures were continued fro 4 h at the shaking rate of 180 rpm.

### Fed-batch cultivations in shake flasks

The fed-batch shake flask cultivations were performed with the gel-based EnBase system in 1 L baffled Erlenmeyer flasks with 200 mL of MSM medium as earlier described [8].

Glucose release for substrate limited growth was generated by 12 AGU L^-1 ^in the cultivation medium. Product synthesis in the selected expression platforms was induced at OD_600 _= 5 ± 0.5 (μ ≈ 0.22 h^-1^). Induction was performed by addition of IPTG to a final concentration of 0.2 mM. The necessary amount of DTT was added as dry powder to the cultivation medium to achieve a final concentration of 12 mM. After induction the cultures were continued for 4 h at 22°C at a shaking rate of 180 rpm.

### Bioreactor processes

Batch and fed-batch cultivations were performed in a 10 L working volume Biostat C bioreactor (B. Braun Biotech, Melsungen, Germany) with the following parameters: the pO_2 _was maintained at 30% by adapting the stirrer rate and automatic regulation of the air flow (from 0 to 30 liters per min), the cultivation temperature before RI induction was 37°C. After induction it was downregulated to 22°C and kept until the end of the process. The pH was controlled at 7.0 ± 0.1 by addition of NH_4_OH (25%) or H_3_PO_4 _(2 M).

The feeding rate was controlled by the Biostat software (version 4.62). The process was monitored by the MFCS/win 2.0 supervisory system. Fed-batch cultivations were started with a volume of 8.0 L of MSM with 15 g L^-1 ^of glucose. Exponential feeding profiles were programmed to maintain a specific growth rate of μ ≈ 0.22 h^-1^. The feeding profiles were calculated with following equations:

where F_o _is the initial feeding rate [L h^-1^], μ is the specific growth rate [h^-1^] to be maintained during feed operation, and t is the time after feed start [h]. The initial feeding rate was calculated from the mass balance on substrate according to

Here, X_0 _and V_0 _are the cell dry weight (CDW) [g L^-1^] and the culture volume [L] at the time of the feeding start, respectively, S_f _[g L^-1^] is the substrate concentration in the feeding solution, and Y_x/s _is the yield coefficient (g CDW per g of glucose). Y_x/s _in all cases (cytoplasmic and periplasmic expression strains) was calculated from batch fermentations as 0.3 g g^-1^.

Before initiation of the fed batch mode cells were cultivated as a batch until OD_600 _≈ 18. RI synthesis was induced during the fed-batch cultivation mode at an OD_600 _of 28, the specific growth rate at the time of induction was the same in all cases (μ = 0.22 h^-1^). The exponential feed function was continued after induction in all fed-batch experiments. Batch cultures were induced at an OD_600 _of 7 (μ ≈0.45 h^-1^).

In all bioreactor synthesis experiments 148 mL of 0.65 M DTT solution were added after 2 h of RI induction to achieve a final concentration of 12 mM in the cultivation medium. In the experiments with DTT feeding the same solution was added repeatedly, starting at 2 hours after RI induction by addition of 148 mL of 0.65 M DTT solution and continued by repeated addition of 74 mL 0.65 DTT solution every following 60 min. At the first DTT addition point the air flow was decreased from 30 to 2-4 L min^-1 ^and the stirrer was manually regulated to maintain 0% of oxygen concentration in the cultivation medium. In all cases the target protein synthesis continued for another 3 hours.

### Analytical tools

Cell samples harvested from flask and bioreactor cultivations were resuspended in lysis buffer with the following biomass to buffer ratio: 1 g of biomass with 10 mL of lysis buffer (50 mM Tris-H_3_PO_4 _pH 8.0, 0.1% Triton X-100, 2 mM EDTA, 1 mM PMSF, 15 mM DTT, 10% propyleneglycol and 0.1 mg mL^-1 ^lysozyme). After 30 min of lysis at +4°C the biomass was sonicated for 60 sec (Vibra cell™, Sonic and Materials Inc., 6 mm diameter probe tip) at 4°C. The soluble and insoluble protein fractions were separated by centrifugation for 30 min, 14000 rpm, 4°C. The total protein fraction represents cellular debris suspension (crude extract) before centrifugation. After centrifugation the insoluble protein pellet was additionally washed and resuspended in the original volume of lysis buffer without lysozyme. The periplasmic protein fractions were extracted by the standard osmotic shock procedure. Therefore after centrifugation the cell pellet was resuspended in 5 mL of ice cold solution, containing 20% (w/v) sucrose, 100 mM Tris-H_3_PO_4 _(pH 8.0), and 0.5 mM Na_2_EDTA. After incubation for a 10 min at +4°C cells were harvested by centrifugation at 10,000 rpm for 10 min and +4°C. After removal of the supernatant the cell pellet was again resuspended in 5 mL of ice cold deionised water, containing 15 mM of DTT. After another incubation for 10 min and centrifugation the supernatant (containing the target protein) was suplemented with 2.0 ml of buffer (250 mM Tris-H_3_PO_4 _pH 8.0, 0.4% Triton X-100, 8 mM EDTA, 4 mM PMSF, 30% propylene glycol).

Samples for SDS-PAGE separation were prepared as follows: 20 μL of protein sample (total soluble, insoluble, protein suspensions), 25 μL of 4×SDS-PAGE loading buffer (Fermentas), 5 μL of 20×DTT (Fermentas) and 50 μL of deionised water to obtain a final sample volume of 100 μL. Samples were heated for 15 min at 95°C. 10 μL of sample was applied to each lane of a 10% SDS-PAGE gel.

The amounts of target were determined from scanned SDS-PAGE gel images with TotalLab software. The gels with separated sample proteins were produced for TotalLab quantifications with internal BSA standards (3 concentration points).

The amount of active RI in the soluble fraction was determined by an activity assay described by Blackburn et al. [28] and is presented in kilo units per gram cell wet weight (kU gCWW^-1^). 1 mg of RI corresponds to 100 kU [28].

The N-terminal amino acid sequence of processed RI was determined by the Edman degradation procedure in Biocentrum Ltd. (Krakov, Poland) from insoluble protein fraction sample after RI production with DTT in the medium and separation on an 8% SDS-PAGE gel.

The amount of oxidized/reduced DTT in the cultivation medium was determined with the Measure-iT™ Thiol Assay kit (Invitrogen), by following the producer's recommendations.

## Competing interests

The authors declare that they have no competing interests.

## Authors' contributions

JS designed the experimental setup, performed all cultivation experiments and prepared the manuscript. PN initiated the project, assisted with data analysis and manuscript preparation. All authors read and approved the final manuscript.
